# Isolation and identification of culturable fungi from the genitals and semen of healthy giant pandas (*Ailuropoda melanoleuca*)

**DOI:** 10.1186/s12917-017-1231-0

**Published:** 2017-11-21

**Authors:** Xiaoping Ma, Changcheng Li, Jiafa Hou, Yu Gu

**Affiliations:** 1 College of Veterinary Medicine, Nanjing Agricultural University, Nanjing, 210095 China; 2 College of Veterinary Medicine, Sichuan Agricultural University, Chengdu, 611130 China; 3 College of Life Sciences, Sichuan Agricultural University, Ya’an, 625014 China

**Keywords:** Culturable fungi, Giant panda (*Ailuropoda melanoleuca*), Vagina, Foreskin, Semen

## Abstract

**Background:**

In order to better understand the possible role of fungi in giant panda reproduction and overall health, it is important to provide a baseline for the normal fungal composition in the reproductive system. Using morphology and internal transcribed spacer (ITS) sequence analysis, we systematically isolated and identified fungal species from the vagina, foreskin, and semen of 21 (11 males and 10 females) healthy giant pandas to understand the normal fungal flora of the genital tracts.

**Results:**

A total of 76 fungal strains were obtained, representing 42 genera and 60 species. Among them 47 fungal strains were obtained from vaginal samples, 24 from foreskins, and 5 from semen samples. Several fungal strains were isolated from more than one sample. More fungal species were isolated from females from males. The predominant genera were *Aspergillus*, *Trichosporon*, and *Penicillium*, followed by *Candida*, *Cladosporium*, *Sordariomycetes*, and *Diaporthe*. The average number of strains in the female vagina was significantly higher than in the foreskin and semen of male.

**Conclusions:**

A total of 60 fungal species (belonging to 42 genera) were identified in the giant panda’s genital tract. Some of the species were commonly shared in both males and females. These findings provide novel information on the fungal community in the reproductive tracts of giant pandas.

## Background

The giant panda is one of the rarest endangered animals [1]. In recent years, breeding giant pandas in captivity has become necessary for conservation and improving their reproductive rate. Although the captive giant panda population has increased, its genetic diversity is far lower than that of the wild population [2], and some captive female giant pandas are unable to carry cubs to term or are subject to secondary infertility.

Microorganisms are responsible for diseases that can directly or indirectly affect reproductive success in animals. Fungi are one of the most important microorganisms that are known to cause abortion in cattle [3] and horses [4].

Fungal infections are becoming more frequent in humans due to the widespread use of antibiotics and immunosuppressors [5]. In 1929, a study confirmed the existence of fungi in the human vagina [6]. *Torulopsis* and *Candida* spp. were later identified from vaginal samples [7].

Studies have confirmed the presence of fungi in the reproductive organs of animals [8–10]. To date, few studies have been performed on microorganisms of the genitourinary tract of the giant panda [11], and fungal infections have been identified as a potential threat to giant panda fertility [12]. Low breeding rates strongly affect the population of giant pandas. There is a close relationship between sterility and vaginal dysbiosis, which is characterized by the imbalance of vaginal microflora due to various disorders and conditions. This highlights the importance of an improved understanding of the normal vaginal flora of giant pandas.

In this study, we investigated the fungal compositions in the vaginas, foreskins, and semen of 21 healthy giant pandas living in a semi-captive, semi-closed breeding environment. Our goal is to provide a baseline information on the normal fungal flora in the reproductive system to better understand the possible roles of fungi in the reproduction and overall health of giant pandas.

## Methods

### Animals

The giant pandas used in this study lived in a semi-captive, semi-closed breeding environment at the Chengdu Research Base of Giant Panda Breeding (Sichuan, China). The pandas were fed bamboo shoots and steamed corn bread. They were allowed to drink water *ad libitum* *.*

Samples were collected from 10 female during estrus and 11 male pandas. Details of the samples are listed in Table 1. None of the animals had previous genital infections noted in their files, and detailed physical examinations found no evidence of vaginitis or balanoposthitis. Pandas with a recent history of disease or animals treated with antifungal drugs during the last 6 months were excluded from this study.

**Table 1:** List of samples taken from giant pandas

Name	Sex	Sample	Age	Sample date	Note
A	female	vaginal secretion	11 yr. 5mo.	2013.4	Pre-procreated
B	female	vaginal secretion	5 yr.	2013.3	First artificial breeding
C	female	vaginal secretion	5 yr. 7mo.	2013.2	First artificial breeding
D	female	vaginal secretion	12 yr.	2013.4	First artificial breeding
E	female	vaginal secretion	6 yr. 9mo.	2013.5	First artificial breeding
F	female	vaginal secretion	7 yr. 8mo.	2013.3	First artificial breeding
G	female	vaginal secretion	5 yr. 7mo.	2013.4	First artificial breeding
H	female	vaginal secretion	–	2013.3	First artificial breeding
I	female	vaginal secretion	18 yr. 8mo.	2013.2	Not pregnant for 5 years
J	female	vaginal secretion	10 yr. 7mo.	2013.4	Not pregnant for 2 years
K	male	prepuce inclusions	10 yr. 3mo.	2013.3	
L	male	prepuce inclusions	14 yr. 7mo.	2013.4	
M	male	prepuce inclusions	10 yr. 7mo.	2013.5	
N	male	prepuce inclusions	10 yr. 7mo.	2013.4	
O	male	prepuce inclusions and semen	10 yr. 6mo.	2013.4	
P	male	semen	8 yr. 6mo.	2013.5	
Q	male	prepuce inclusions	7-10 yr	2013.3	
R	male	prepuce inclusions	8 yr	2013.4	
S	male	prepuce inclusions	7 yr.8mo	2013.2	
T	male	prepuce inclusions	8 yr.	2013.4	
U	male	prepuce inclusions and semen	8 yr. 7mo	2013.4	

Note: Because panda H was captured in the wild, its age is not known

### Sampling procedure

Samples were collected during estrus when the female pandas were ready for artificial insemination from February to May of 2013. All personnel involved in sampling wore sterile protective clothing, hats, masks, and latex gloves. Ketamine was used for anesthesia in giant pandas, and isoflurane was administered via anesthetic machine to maintain anesthesia. Samples were collected from either the bottoms of vaginas (cervical orifice) or foreskins with sterilized cotton swabs when the pandas were supine after anesthesia. The vulvas, foreskins, and surrounding areas were rinsed three times with sterilized, warm physiological saline solution, then wiped with a disposable sterile towel. Samples were collected using guarded swabs by inserting a sterile cervical canal into the cervical. Sterile cotton swabs were then inserted to the vagina bottoms (cervical orifice) through the cervical canal to collect the cervix outflow. The semen from three male giant pandas was collected through electroejaculation after general anesthesia, with sterilized cotton swabs using a sterilized special semen collection cup. Special care was taken to ensure that the swab did not come into contact with other parts of the body. All samples were quickly placed in sterilized plastic sample bags, transported into laboratory on ice within 2 h, and then immediately inoculated under a BSL-2 safety cabinet. A total of 23 samples were subjected to fungal analysis (Table 1).

### Fungal culture and identification

#### Fungal culture

Samples were streak inoculated aerobically onto Sabouraud dextrose agar (MOLTOX, Inc., Boone, NC) containing 2% (*v/v*) sterile olive oil, malt extract agar, or yeast extract peptone dextrose agar. All media were supplemented with antibiotics (Chloramphenicol 0.005% (m/v)).

Cultural examination was carried out in a BSL-2 safety cabinet of a bioclean room. Sterilized sealing film was used to cover each plate. Blank plates were used as control to ensure no microorganisms were from aerial contamination. Each sample was plated in 3 culture plates with 3 control plates. All culture dishes were inoculated and stored at 25 °C for 7–30 days before being considered negative. As soon as a new fungal colony appeared, the colony was picked and planted in another dish containing the same medium for cultivation.

#### Morphology identification

Fungal morphology was determined according to previous procedures [13].

#### Genetic analysis

Genomic DNA of the isolates was individually extracted using a Yeast DNA Kit (Tiangen Biotech Co., Ltd., Beijing, China) and stored at −20 °C before further analysis.

Fungal DNA amplification was performed using the primer set of an ITS located between ITS1 to ITS4 that was specific for a ribosomal DNA gene: forward, 5′-TCCGTAGGTGAACCTGCG-3′; reverse, 5′-TCCTCCGCTTATTGATATGC-3′. PCR amplification was performed in a 50-μl reaction mixture containing 19 μl 2 × Taq Master Mix (Tiangen Biotech Co., Ltd., Beijing, China), 2 μl primers, 25 μl ddH2O, and 2 μl of fungal genomic DNA. The thermocycling conditions were as follows: 5 min at 94 °C (initial denaturation), 30 cycles of 45 s at 94 °C, 45 s at 58 °C, 72 °C extension for 60 s, cycle number 40, and a final extension of 7 min at 72 °C. The PCR products (8 μl) were examined using 1.0% agarose gel electrophoresis containing 0.5 mg/ml of ethidium bromide. A 300–600 bp fragment of the rDNA was produced. The PCR products were subjected to DNA sequencing performed by Invitrogen (Shanghai Invitrogen Biotechnology Company, Shanghai, China).

Partial gene sequences of the isolates were submitted to GenBank Accession and the numbers are listed in Table 2. A sequence similarity search was performed using BLAST (https://www.ncbi.nlm.nih.gov/nuccore/). Isolates were identified on the selection of the most similar sequences using BLAST. The phylogenetic tree was constructed by Mega 6.0 software with neighbor-joining method.

**Table 2:** Fungal strains identified in vaginal secretion, foreskin inclusion, and semen of giant pandas

Name	Gender	Strain No. in sample	Sample type	Fungal taxon	GenBank accession number
A	Female	1	VS	*Candida catenulate*	KF973198
H	Female	3	VS	*Pseudozyma aphidis*	KF973199
				*Arthrinium sacchari*	KF973200
				*Trichoderma* sp.LCC02–03	KJ531959
J	Female	3	VS	*Diaporthe melonis*	KF986543
				*Myrmecridium schulzeri*	KF986544
				*Lecythophora* sp.LCC06	KF986545
D	Female	5	VS	*Cladosporium cladosporioides*	KF986546
				*Chaetomium globosum*	KF986547
				*Aspergillus niger*	KF986548
				*Sordariomycetes* sp.LCC10	KF986549
				*Dicyma pulvinat*	KF986550
E	Female	4	VS	*Aspergillus versicolor*	KF986551
				*Aureobasidium pullulans*	KF986552
				*Diaporthe phaseolorum*	KF986553
				*Fusarium proliferatum*	KF986554
F	Female	6	VS	*Trichosporon guehoae*	KF990133
				*Penicillium marneffe*	KF990134
				*Penicillium commune*	KF990135
				*Botryotinia fuckeliana*	KF990136
				*Gliocladium roseum*	KF990137
				*Bjerkandera adusta*	KJ152158
G	Female	7	VS	*Pestalotiopsis vismiae*	KF990138
				*Thanatephorus cucumeris*	KF990139
				*Fusarium merismoides*	KF990140
				*Debaryomyces hansenii*	KF990141
				*Candida catenulata*	KF990142
				*Pseudocercosporella fraxini*	KF990143
				*Xylariaceae* sp.LCC28	KF990144
B	Female	2	VS	*Penicillium marneffei*	KF990145
				*Phanerochaete sordid*	KF990146
I	Female	14	VS	*Isaria farinose*	KF990147
				*Penicillium digitatum*	KF990148
				*Penicillium brevicompactum*	KF990149
				*Aspergillus sclerotiorum*	KF990150
				*Alternaria alternata*	KF990151
				*Aspergillus ruber*	KF990152
				*Diaporthe phaseolorum*	KF990153
				*Ascomycota* sp.LCC38	KF990154
				*Epicoccum nigrum*	KF990155
				*Basidiomycota* sp.LCC40	KF990156
				*Plectosphaerella cucumerin*	KF990157
				*Trichosporon japonicum*	KF990158
				*Trichosporon brassicae*	KF990159
				*Trichosporon cutaneum*	KF990160
C	Female	2	VS	*Aspergillus versicolor*	KJ531956
				*Bjerkandera adusta*	KJ531957
K	Male	1	FI	*Alternaria alternata*	KC896385
L	Male	1	FI	*Candida catenulata*	KJ152159
O	Male	1	FI	*Sordariomycetes* sp.LCC03–1	KJ531958
T	Male	4	FI	*Aspergillus fumigatus*	KJ531960
				*Cladosporium cladosporioides*	KJ531961
				*Trichosporon asteroides*	KJ531962
				*Meyerozyma guilliermondii*	KJ531963
M	Male	1	FI	*Meyerozyma guilliermondii*	KJ152160
S	Male	4	FI	*Aspergillus versicolor*	KJ152161
				*Schizophyllum commune*	KJ152162
				*Rhizoctonia solani*	KJ152163
				*Trichosporon japonicum*	KJ152164
N	Male	3	FI	*Plectosphaerella cucumerina*	KJ152165
				*Beauveria bassiana*	KJ152166
				*Sordariomycetes* sp.LCC07–3	KJ152167
Q	Male	7	FI	*Cladosporium tenuissimum*	KJ152168
				*Ceriporia lacerate*	KJ152169
				*Colletotrichum gloeosporioides*	KJ152170
				*Tritirachium* sp.LCC08–4	KJ152171
				*Trichosporon jirovecii*	KJ152172
				*Nigrospora* sp.LCC08–6	KJ152173
				*Aspergillus versicolor*	KJ152174
R	Male	2	FI	*Arthrinium phaeospermum*	KJ152175
				*Periconia byssoides*	KJ152176
U	Male	0	FI	–	–
O	Male	2	SE	*Trametes versicolor*	KJ531965
				*Leptosphaeria* sp.LCC1–2	KJ531966
P	Male	2	SE	*Thanatephorus cucumeris*	KJ531967
				*Chaetomium globosum*	KJ531968
	Male	1	SE	*Trametes versicolor*	KJ531969

Note: in the sample type, “VS” represents vaginal secretion, “FI” represent foreskin inclusion, and “SE” represents semen

### Statistical analysis

Data were analysized by IBM SPSS 20.0 software for Windows. The independent-samples test of nonparametric tests or one-way ANOVA were used. Differences showing *p*-values less than 0.05 were considered statistically significant.

## Results

### Morphological identification

In this study, a total of 23 samples from vaginas, foreskins, and semen were collected from 21 healthy giant pandas (Table 1). Fungi were isolated from 22 samples, and only one sample (foreskin inclusions of panda U, Table 1) was found free of any fungus. The fungi were identified either as multicellular filaments (mycetes) or single-celled yeasts. Mycetes were isolated from 9 of the 10 vaginal samples, 7 of the 10 foreskin inclusions, and 3 of the 3 semen samples. Yeasts were isolated from 5 of the 10 vaginal samples, 6 of the 10 foreskin inclusions, and none of the semen samples (Table 2).

Morphological identification revealed 82 total isolates, including 51, 26, and 5 from the vagina, foreskin, and semen samples, respectively.

### Genetic identification

**Fig. 1:**
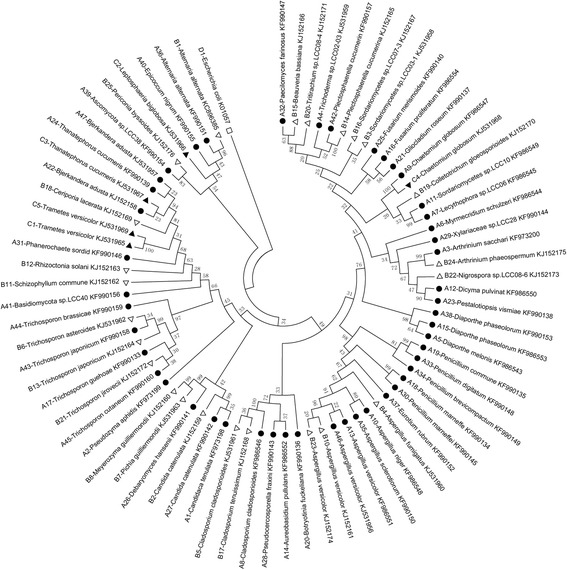
Neighbor-joining phylogenic tree of 76 strains isolated from each sample. Solid circles (●) indicate strains isolated from vaginas; hollow triangles (∆) indicate strains isolated from foreskins; solid triangles (▲) indicate strains isolated from semen; hollow squares (□) indicate reference sequences from GenBank

The 82 fungal isolates were sequenced and analyzed by NCBI Blast. The nucleotide sequence and blast results showed that several isolates had nearly the same base sequence; therefore, these were identified as the same strain. Finally, 76 fungi strains were obtained, and a phylogenetic tree was constructed (Fig. 1). Forty-seven strains were identified from the 51 isolates in vaginal samples, 24 strains from the 26 isolates in foreskin samples, and 5 strains from the 5 isolates in semen samples (Table 2, Fig. 1).

### Analysis of strain composition

#### Analysis of strains at the species level

Several species, such as *Alternaria alternate, Aspergillus versicolor*, and *Candida catenulate*, were present in more than one sample. After removing identical strains in the samples, 60 fungal species were identified in the giant panda’s genital tract. Several species, including *A. alternate, A. versicolor, C. catenulate, Chaetomium globosum, Cladosporium cladosporioides, Plectosphaerella cucumerina, Thanatephorus cucumeris*, and *Trichosporon japonicum*, were isolated from both male and female giant pandas.

The 47 strains in the vaginal samples were identified as 42 species; the 24 strains in the foreskin samples were identified as 22 species; and the 5 strains in the semen samples were identified as 4 species (Table 2).

#### Analysis of strains at the genus level

The 76 strains belong to 42 genera. The predominant genera were *Aspergillus* (10.53%, 8/76), *Trichosporon* (9.21%, 7/76), and *Penicillium* (6.58%, 5/76). *Cladosporium, Diaporthe*, and *Sordariomycetes* accounted for 3.95% (3/76), while *Alternaria*, *Arthrinium*, *Bjerkandera*, *Chaetomium*, *Fusarium*, *Meyerozyma*, *Plectosphaerella*, *Thanatephorus*, and *Trametes* accounted for 2.63% (2/76). The other genera were represented by one strain each (1/76) (Table 3).

**Table 3:** Genus distribution of all strains

Genus level	strain No.	percentage of all strains (%)	Vaginal No.	Foreskin No.	Semen No.
*Aspergillus*	8	10.53	5	3	0
*Trichosporon*	7	9.21	4	3	0
*Penicillium*	5	6.58	5	0	0
*Candida*	3	3.95	2	1	0
*Cladosporium*	3	3.95	1	2	0
*Diaporthe*	3	3.95	3	0	0
*Sordariomycetes*	3	3.95	1	2	0
*Alternaria*	2	2.63	1	1	0
*Arthrinium*	2	2.63	1	1	0
*Bjerkandera*	2	2.63	2	0	0
*Chaetomium*	2	2.63	1	0	1
*Fusarium*	2	2.63	2	0	0
*Meyerozyma*	2	2.63	0	2	0
*Plectosphaerella*	2	2.63	1	1	0
*Thanatephorus*	2	2.63	1	0	1
*Trametes*	2	2.63	0	0	2
*Ascomycota*	1	1.32	1	0	0
*Aureobasidium*	1	1.32	1	0	0
*Basidiomycota*	1	1.32	1	0	0
*Beauveria*	1	1.32	0	1	0
*Gliocladium*	1	1.32	1	0	0
*Botryotinia*	1	1.32	1	0	0
*Ceriporia*	1	1.32	0	1	0
*Colletotrichum*	1	1.32	0	1	0
*Debaryomyces*	1	1.32	1	0	0
*Dicyma*	1	1.32	1	0	0
*Epicoccum*	1	1.32	1	0	0
*Isaria*	1	1.32	1	0	0
*Lecythophora*	1	1.32	1	0	0
*Leptosphaeria*	1	1.32	0	0	1
*Myrmecridium*	1	1.32	1	0	0
*Nigrospora*	1	1.32	0	1	0
*Periconia*	1	1.32	0	1	0
*Pestalotiopsis*	1	1.32	1	0	0
*Phanerochaete*	1	1.32	1	0	0
*Pseudocercosporella*	1	1.32	1	0	0
*Pseudozyma*	1	1.32	1	0	0
*Rhizoctonia*	1	1.32	0	1	0
*Schizophyllum*	1	1.32	0	1	0
*Tritirachium*	1	1.32	0	1	0
*Xylariaceae*	1	1.32	1	0	0
*Trichoderma*	1	1.32	1	0	0

#### Comparison between the proportion of samples with mycetes and with yeasts

**Fig. 2:**
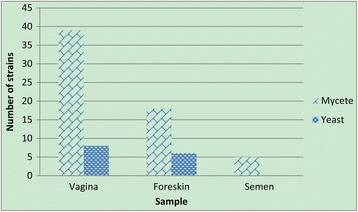
Prevalence of mycetes and yeasts isolated from different types of samples

A total of 47 different strains were found in the vaginal samples, including 39 mycetes (51.32%, 39/76) and 8 yeasts (10.53%, 8/76). A total of 24 different strains were found in the foreskin samples, including 18 mycetes (23.68%, 18/76) and 6 yeasts (7.89%, 6/76). A total of 5 mycetes (6.58%, 5/76) were isolated in semen samples. In brief, among the 76 strains, the number of mycetes (62) was significantly higher than the number of yeasts (14) in all three sample types (*p = 0.0002*) (Fig. 2, Table 2).

### Differences in strain number distribution among the three sample types

The number of strains and the range of variation were compared among the three sample types. A large range of strain numbers, from 1 to 14, was found in the vaginal samples. However, only 1 sample was found to have 14 strains; the remaining samples had 2~7 strains. A total of 24 strains were isolated from the foreskin samples, ranging from 0 to 7 per animal. Only 1 sample was found to have 7 strains, and 1 sample had no fungus. The others had 1~4 strains. In the 3 semen samples, only 5 strains (with 1, 2, and 2 strains) were isolated. Our results indicate that the number of fungi in the genital tract was significantly higher in the female pandas than in the male pandas (*p = 0.042*). (Table 2).

Regarding the number of strains from the three sample types, we found that the vaginal secretion had the highest number of fungal strains with an average of 5.67 isolates per animal. The average strains from foreskin inclusion and semen were 3.71 and 1.57, respectively. Additionally, several species, including *Fusarium* spp. and *Penicillium* spp., were present in multiple female samples; *Meyerozyma guilliermondii* and *Trametes versicolor* were present in multiple male pandas.

### Analysis of variation of fungal constitution between pregnant and nonpregnant female pandas

Of the 10 female pandas, 4 had already undergone successful pregnancy and 6 had never undergone successful breeding. There were distinct differences in the strains and species between these two groups. There were 1, 2, 2, and 5 strains in the vaginas of the pregnant pandas and 3, 3, 4, 6, 7, and 14 strains in the non-pregnant pandas. The average number of strains in the nonpregnant group (average: 6.2 strains) was higher than in the pregnant group (average: 2.5 strains), but the difference was not statistically significant (*p = 0.054*) (Tables 1, 2). Whether the high number of fungi in the vagina is related to non-pregnancy remains unclear.

The composition of fungal species between the pregnant and nonpregnant pandas was analyzed. There were 42 species in female pandas, and only a few species (4/42) were common between the two groups, including *A. versicolor*, *Bjerkandera adusta*, *C. catenulate*, and *Penicillium marneffei*. All other species(38/42)differed between these two groups.

### Analysis of fungal constitution between younger and older pandas

To determine whether fungal constitution is related to age, the female giant pandas were divided into an older (age > 10 years, 4 pandas) and younger group (age < 10 years, 6 pandas). The average strain count was 4.0 per animal for the younger group and 5.75 per animal for the older group. There were 1, 3, 5, and 14 strains in the older group, and 2, 2, 3, 4, 6, and 7 strains in the younger group. However, there were no significant differences between the older and younger groups according to the ANOVA test (*p = 0.505*). Considering the composition of fungi between the older and younger female pandas, only 5 common genera were found between the two groups, including *Aspergillus*, *Candida*, *Penicillium*, *Diaporthe*, and *Trichosporon*. The other 26 genera differed (Tables 1, 2).

The 10 male pandas also were divided into an older (age > 10 years, 5 pandas) and younger group (age < 10 years, 5 pandas). There were 1, 1, 1, 2, and 3 strains from the foreskin of the older group and 0, 3, 4, 4 and 7 strains from the foreskin of the younger group. The younger pandas (average 3.6 fungal strains) had more fungal strains than the older pandas (average 1.6 fungal strains). Due to the small sample size, this difference was not statistically significant (*p = 0.151*). However, the composition of fungal species differed between the older and younger male pandas, with the exception of the strain *Meyerozyma guilliermondii*, which was present in both an 8-year-old (T) and a 10-year-old (M) (Tables 1, 2).

## Discussion

### The fungal diversity of the giant panda genitals and semen

Previous studies performed isolation and identification of bacteria from the vaginas, foreskins, and semen of giant pandas [11, 14]. This study investigated the fungal flora in these samples. A total of 76 culturable fungal strains were identified. Our data indicates that the fungi are common in captive giant pandas (in 100% of the examined vaginal and semen samples, and in 90% of the examined foreskin samples). Our results show that there were significantly more mycete species (64/76) than yeasts (12/76) in the vaginas, foreskins, and semen of giant pandas. The mycetes were 5-fold higher than yeasts in the vaginas and 2.67-fold higher in the foreskins. However, no yeast was found in semen, which is similar to a previous report showing no yeast in the semen of 11 healthy male stallions [10].

### Normal fungal species in the reproductive tract of the giant panda

Several mycete species are considered to be pathogenic in humans or other animals [15]. The most common species are *Aspergillus* and *Penicillium*, which are ubiquitous fungi, usually found as saprophytes. The significance of *Aspergillums* and *Penicillium* in mammalian disease is heightened by their production of potent mycotoxins. *P. marneffei* is reported to be pathogenic to Vietnamese bamboo rats [16] and humans [17]. Annam reported that *P. marneffei* caused genital ulcers in an HIV-infected patient [18]. However, no studies have shown *P. marneffei* to cause disease in giant pandas. In this study, *P. marneffei* was isolated from both breeding and non-breeding female pandas.

In this study, *A. fumigatus* was isolated from the foreskin inclusion of a healthy male panda, and *A. niger* was isolated from the vaginal secretion of a healthy female panda that had been successfully bred. *Aspergillus* spp. has also been reported to be associated with reproductive disorders. *Aspergillus* was responsible for mycotic abortion in cattle [3]. *A. fumigatus* was the most frequent isolate (62%) in bovine mycotic abortion [19], and *A. niger* is the most common isolate in 120 bovine animals that have had clinical histories of reproductive disorders [20]. In this study, however, *A. fumigatus* was isolated from the foreskin inclusion of a healthy male panda and *A. niger* from the vaginal secretion of a healthy female panda that had been successfully bred. It is unlikely that these *Aspergillus* spp. cause miscarriage or other reproductive disorders in giant pandas.

*Cladosporium cladosporioides*, which causes reproductive disorders in cows [20] and phaeohyphomycosis in humans [21] and giant pandas [12], was also isolated from the vagina and foreskin samples.

The microbial flora of giant pandas are significantly different from those of other animals due to their unique diet and habitat [22]. The identification of these fungal species from the healthy pandas suggests that they may be part of the normal fungal flora of the reproductive tract and that they may serve as a barrier against infectious agents [14]. These species may otherwise cause diseases when they reach and grow in locations different from the reproductive tract [23].

### The yeast species in the reproductive tract of giant pandas

In this study, we found fewer yeast species, and none were cultured from semen, suggesting that yeasts may be less culturable. Our results showed that 20% of the isolated fungi consisted of yeast, including one species of *Candida* and six species of *Trichosporon*. *Candida spp.* often cause mucocutaneous syndromes and invasive candidiasis in certain settings [24], suggesting that they may be genital tract (mucosas system) pathogens. *Trichosporon spp.* are considered the second most common agent of yeast-disseminated infections [25]. In this study, *Trichosporon* was found in multiple pandas. Three species, including *T. japonicum*, *T. brassicae*, and *T. cutaneum*, were identified in one panda that had years of infertility. Whether *Trichosporon* can harm the vaginas and foreskins of giant pandas, or whether they are part of the normal fungal flora of the reproductive tract, warrants further study.

### Fungi isolated from an older female panda

Older women are reported to be more susceptible to mycotic vaginitis [26]. Interestingly, 14 strains were isolated from the 18-year-old giant panda named “I”, which had remained nonpregnant for the 5 preceding years. This is significantly higher than the average strains isolated from the female pandas (5.67 per animal). Although inconclusive due to the small sample size, it would be valuable to determine whether the number of fungi correlates with the age of giant pandas, which typically live approximately 20 years in the wild and approximately 30 years in captivity.

### The difference of fungi between pregnant and non-pregnant groups

No significant difference was found in species number between pregnant and non-pregnant groups. This may be due to the small sample size. A larger sample size, although difficult to obtain with giant pandas, would greatly help better understand the possible difference.

Study of the dominance index of species in humans [27] suggests that the dominance index change may lead to reproductive barriers. Further study of the role of dominance index and different fungi species, along with larger sample sizes, may help uncover the role of fungi in panda breeding and health.

## Conclusion

This study identified a total of 76 fungal strains, representing 42 genera and 60 species, from the gentital tracts (vagina, foreskin) and semen of healthy giant pandas. These fungal strains likely represent the normal fungal flora in their reproductive systems. These findings provide previsouly unavialble information on the fungal community, which will facilitate our understanding of the roles of fungi in the overall health and reproduction of giant pandas.
